# Downregulation of Sostdc1 in Testicular Sertoli Cells is Prerequisite for Onset of Robust Spermatogenesis at Puberty

**DOI:** 10.1038/s41598-019-47930-x

**Published:** 2019-08-07

**Authors:** Bhola Shankar Pradhan, Indrashis Bhattacharya, Rajesh Sarkar, Subeer S. Majumdar

**Affiliations:** 1 Cellular Endocrinology Laboratory, National Institute of Immunology, Aruna Asaf Ali Marg, JNU complex, New Delhi, 110067 India; 2 Present Address: Department of Zoology and Biotechnology, HNB Garhwal University, Srinagar, 246174 Pauri Garhwal, Uttarakhand India; 3 National Institute of Animal Biotechnology, Hyderabad, Telangana 500 049 India

**Keywords:** Cell biology, Developmental biology, Differentiation

## Abstract

An alarming decline in sperm count of men from several countries has become a major concern for the world community. Hormones act on testicular Sertoli cells (Sc) to regulate male fertility by governing the division and differentiation of germ cells (Gc). However, there is a limited knowledge about Sc specific gene(s) regulating the spermatogenic output of the testis. Sclerostin domain-containing 1 protein (*Sostdc1*) is a dual BMP/Wnt regulator is predominantly expressed in the Sc of infant testes which hardly show any sign of spermatogenesis. In order to investigate the role of *Sostdc1* in spermatogenic regulation, we have generated transgenic (Tg) rats which induced persistent expression of *Sostdc1* in mature Sc causing reduced sperm counts. Although Sc specific *Sostdc1* did not affect the function of either Sc or Leydig cells (Lc) in the adult testis of Tg rat, we observed a selective augmentation of the BMP target genes via activated phospho smad 1/5/8 signaling in Gc leading to apoptosis. Here, for the first time, we have demonstrated that *Sostdc1* is a negative regulator of spermatogenesis, and provided substantial evidence that down regulation of *Sostdc1* during puberty is critically essential for quantitatively and qualitatively normal spermatogenesis governing male fertility.

## Introduction

About 7% of the world’s male population is infertile, making it a public concern^1–4^. Around one-third of these cases are idiopathic in nature^2,3^ and are not amenable to cure with any of the conventional modes of therapy including hormonal supplementations^5–7^. Therefore, there is an urgent need to determine the cellular causes of such male infertility other than the hormonal factors.

Spermatogenesis is a complex process occurring inside a specialized microenvironment of testis, where various autocrine, paracrine, and endocrine interactions play an important role^8,9^. Sertoli cells (Sc) are the major somatic component of the testis which regulate the division, differentiation and survival of the male germ cells (Gc) into sperm^8^. Postnatal maturation of Sc during pubertal testicular development becomes critical for the robust onset of spermatogenesis^10^. Recent reports suggest that Sc remain immature and show poor response towards hormonal therapy in some patients with idiopathic male infertility^10^.

In neonatal rats (5 days old), spermatogenesis remains in a quiescent state as immature Sc fail to support the robust differentiation of spermatogonial cells^11^. We have recently reported the differential gene expression profile of rat Sc obtained from immature (5 days old) and maturing (12 days old) testes (GSE48795)^11–14^. From this study, we have observed that Sclerostin domain-containing 1 protein (*Sostdc1*), a dual Wnt/BMP regulator^15–18^, was up-regulated in the immature Sc. Conversely, its expression was found to be down-regulated during pubertal maturation of Sc which is associated with robust onset of spermatogenesis.

Increasing evidences indicate both Wnt and BMP signaling play an essential role in the process of sperm production^19–23^. Dysregulation of Wnt/β catenin signaling in either Sc or in Gc leads to impaired spermatogenesis^20,24,25^. The genes related to BMP families, such as *Bmp2*, *Bmp4*, *Bmp7*, *Bmp8a* and *Bmp8b* are known to regulate Gc proliferation, differentiation and survival^26,27^. Interestingly, the activity of *Sostdc1* is mainly regulated by its localization. For example, *Sostdc1* retained in the endoplasmic reticulum (intracellular) inhibits Wnt signaling by reducing cell surface expression of LRP6^28^. On the other hand, secreted form of *Sostdc1* either enhances or inhibits the Wnt signaling depending on the ligand. Similarly, *Sostdc1* has been shown to regulate BMP pathway either positively or negatively depending on its localization in different cell systems. For example, in tooth and kidney, *Sostdc1* inhibits BMP signaling^28–30^ whereas, in the pancreas, it activates the BMP signaling without affecting the Wnt pathway^31^. *Sostdc1* homozygous knock out mice (in both C57BL6 and 129 Sv/EV strains) are viable and fertile, however in the 129 Sv/EV strain litter sizes are often reduced, and there is a frequent increase in the number of resorptions^32^. However, the role of *Sostdc1* in testis in regulating spermatogenesis is not known yet.

Therefore, to delineate any association between the inverse expression of *Sostdc1* and spermatogenic onset, we here have prevented the natural down-regulation of *Sostdc1* expression specifically in pubertal Sc *in vivo*. In this transgenic (Tg) rat model, *Sostdc1* was continued to express in Sc from puberty up to adulthood, which showed an androgen independent, non-obstructive oligozoospermia, and this effect appears to involve the dysregulation of BMP signaling resulting in Gc apoptosis in the Tg testis.

## Results

### *Sostdc1* was predominantly expressed in Sc of immature rat testis

**Figure 1:**
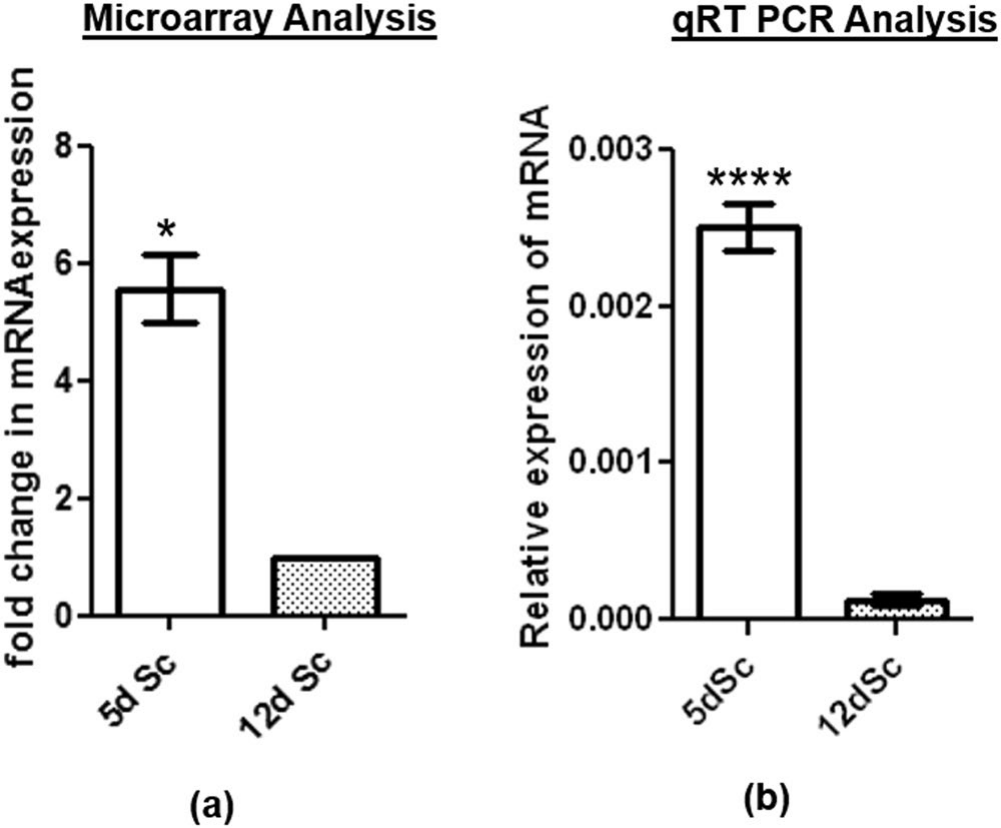
*Sostdc1* was expressed maximally in immature rat testes. (**a**) The expression of *Sostdc1* mRNA level in the Sc of 12 days old rat testis as compared to 5 days old rat testis as revealed in the microarray data analysis. (**b**) Relative expression of *Sostdc1* mRNA level in the Sc of 5 days old rat testes as compared to 12 days old rat testes as evaluated by q-RT- PCR. *Ppia* was the housekeeping gene used as the internal control, *P ≤ 0.05.

We have recently reported a differential microarray analysis (GSE48795)^14^ performed between immature Sc, isolated from spermatogenically quiescent (5 days old) testes and maturing Sc, isolated from spermatogenically active (12 days old) testes of rats^14^. From this study, we have observed that the expression of *Sostdc1* was significantly down-regulated in 12 days old Sc as compared to that of the 5 days old Sc (Fig. 1a). This pattern observed in the array data was further confirmed by the qRT-PCR analysis in three different sets of cultured Sc obtained from these two age groups (Fig. 1b).

### Generation of transgenic (Tg) rats expressing *Sostdc1* in pubertal Sc

Since, *Sostdc1* expression was found to be down-regulated with the pubertal onset of spermatogenesis, we therefore, hypothesized that a persistent expression of *Sostdc1* in Sc during and after puberty may have a critical impact on male fertility. To investigate this hypothesis, we here have prevented such natural down-regulation of *Sostdc1* specifically in Sc from puberty onwards in a novel transgenic (Tg) rat model, and further examined its effect on male fertility during adulthood. Since the proximal Rhox-5 promoter drives the expression of target gene only in Sc from 14 days postnatal age onwards^33^, the full-length open reading frame (ORF) of *Sostdc1* was cloned downstream of proximal Rhox-5 promoter (Supplementary Fig. S1a,b). Using this construct, we generated the Tg rats, and transgene positive pups were screened and confirmed by both PCR and Southern Blot analyses (Supplementary Figs S1c and S2a). The mRNA and protein level expressions of the transgene were validated further by both the semi-quantitative RT-PCR and quantitative RT-PCR analysis (Supplementary Fig. S2b,c) and western blot analyses (Supplementary Fig. S2d). The immuno-histochemical analysis with flag tag antibody in the testicular cross section of 20 weeks old Tg rat testes showed that the Sostdc1 protein was exclusively expressed only in the cytoplasm of Sc (Supplementary Fig. S2e). Testicular sections of 20 weeks old non- transgenic age-matched wild-type (WT) controls did not show any specific immuno-staining. All these data together confirmed that *Sostdc1* was continued to express exclusively in the Sc of the adult Tg testis.

### Spermatogenesis was impaired in the transgenic rats with persistent *Sostdc1* expression

**Figure 2:**
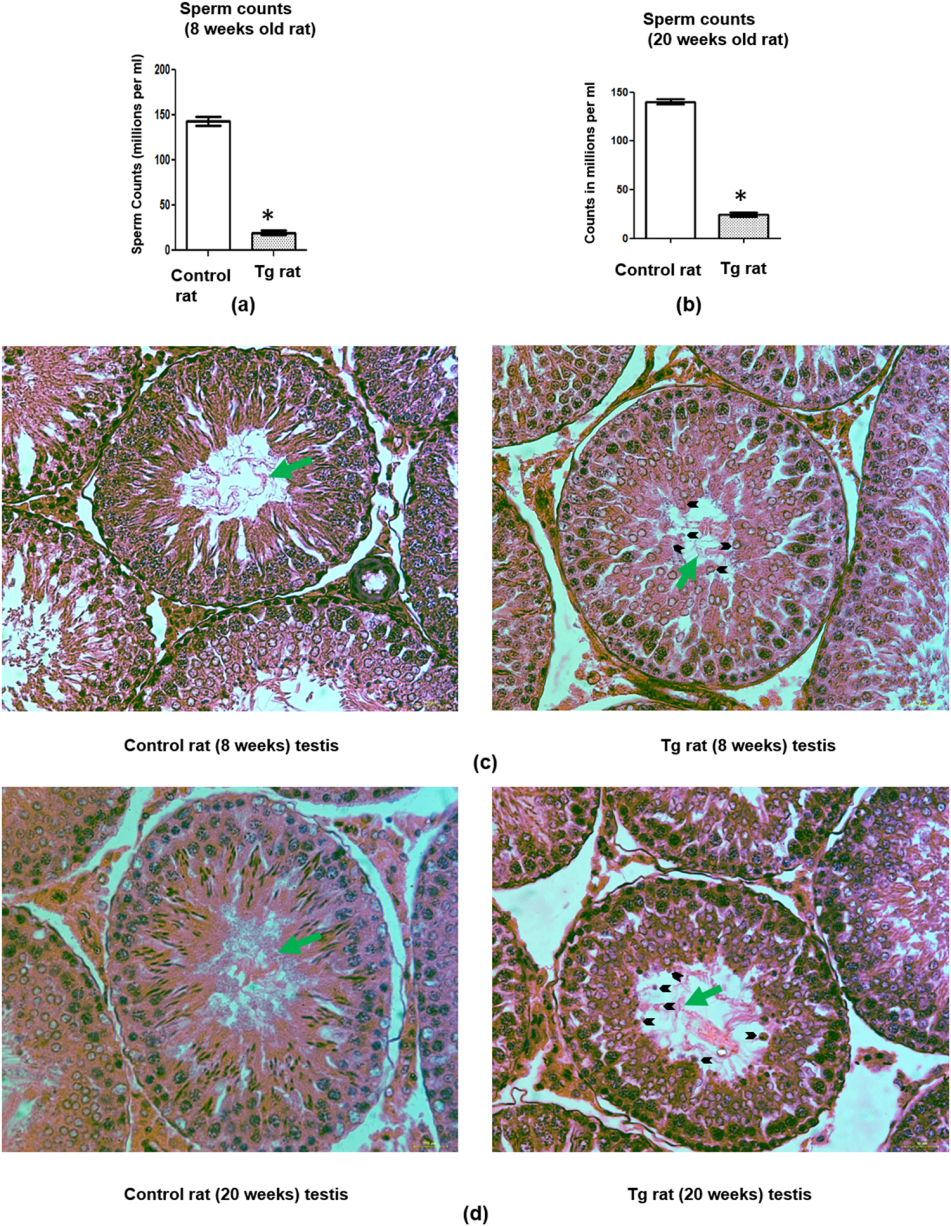
Spermatogenesis was impaired in the transgenic (Tg) rats expressing *Sostdc1* post-pubertally in Sc. (**a**) The caudal epididymal sperm counts in the Tg rats (8 weeks old rat) and that of the age matched wild-type (WT) controls, *P ≤ 0.05; significant difference in relation to control. n ≥ 7. (**b**) The caudal epididymal sperm counts in the Tg rats (20 weeks old rat) and that of age matched WT controls, *P ≤ 0.05; significant difference in relation to control. n ≥ 7. (**c**) Haematoxylin - eosin staining of testis of Tg rat (8 weeks old rat) demonstrating low sperm density in the lumen (green arrow) as compared to that of age matched wild-type (WT) control rat testis and the sloughing up of Gc (black arrow head) were clearly observed in the Tg rat testis. All images were taken at a total magnification of 40X. (**d**) Haematoxylin - eosin staining of testis of Tg rat (20 weeks old rat) demonstrating low sperm density in the lumen (green arrow) as compared to that of age matched WT control rat testis and the sloughing up of Gc (black arrow head) were clearly observed in the Tg rat testis. All images were taken at a total magnification of 40X. Kindly note that the 20X magnification images of haematoxylin - eosin staining of testis of Tg rat (20 weeks old) and age matched WT control have been shown in Supplementary Fig. S4.

*Sostdc1* expressing F_1_ generation adult males (n = 5) were found to be infertile when mated with normal mature WT females (n = 10) for more than three months (for each individual mating set) (Table 1). Therefore, we crossed the *Sostdc1* expressing F_1_ females with WT males to generate F_2_ Tg males on which all the subsequent studies were performed. The testicular histology and other fertility parameters of these F_2_ Tg males were comparable with that of the F_1_ Tg males (Supplementary Figs S3, S4 and Fig. 2). It is essential here to note that the libido of the F_1_ and F_2_ Tg rats were found to be intact as detected the copulatory vaginal plugs in all the wild type (WT) females mated with Tg males (both of F_1_ and F_2_ generations).

**Table 1:** Fertility status of males and females of transgenic rats.

Groups	Breeding pair	Vaginal Plug Detected	Total number of pups (Litter size)	Fertility
*WT males* *X* *WT females*	5 (1 male and 2 females)	Yes	126	Fertile
*F*_1_ *Tg males* *X* *WT females*	5 (1 male and 2 females)	Yes	0	Infertile
*WT males* *X* *F*_1_ *Tg females*	5 (2 females and 1 male)	Yes	118	Fertile
*F*_2_ *Tg males* (*generated by crossing F*_1_ *Tg female with WT males*) *X* *WT females*	5 (1 male and 2 females)	Yes	0	Infertile

To determine the effect of such persistent *Sostdc1* expression in Sc on spermatogenic initiation and maintenance, we have carefully analysed the different fertility parameters in Tg rats in an age-specific manner (e.g. 8 weeks and 20 weeks of age, respectively). At 8 weeks of age, there was a significant (p ≤ 0.05) reduction in the sperm counts in Tg rats (20 ± 0.05 × 10^6^/ml/epididymis) as compared to age-matched WT controls (148 ± 0.37 × 10^6^/ml/epididymis) (Fig. 2a). Although total sperm count was observed to be drastically reduced, more than 90% of the sperm were detected to be motile in Tg rats (data not shown). The testes of 8 weeks old Tg rats showed sloughing of spermatogenic cells (black arrow heads) in the tubules. The sperm density (green arrow) was very less in the lumen of seminiferous tubules. These tubular abnormalities were rarely detected in testis of age-matched WT controls (Fig. 2c). These data suggested that long term expression of *Sostdc1* may have affected the first wave of spermatogenesis. A similar fertility defect was also observed in the Tg rat at 20 weeks of age. There was also a drastic reduction in sperm count in the testis of Tg rat at this age (Figs 2b,d and S4). All these results together suggested that uninterrupted expression of *Sostdc1* in Sc affect sperm production in adult rat testes. Such a drastic reduction in sperm count (86%) may be the principle cause of oligozoospermia with male infertility as observed in our Tg rats. Oligozoospermic mutant mouse models for several genes with around 70–90% reduction in sperm count have been reported previously with complete male infertility supporting our present observation^34,35^.

### Effect of long term Sostdc1 expression on testicular cells

#### Leydig cells (Lc)

**Figure 3:**
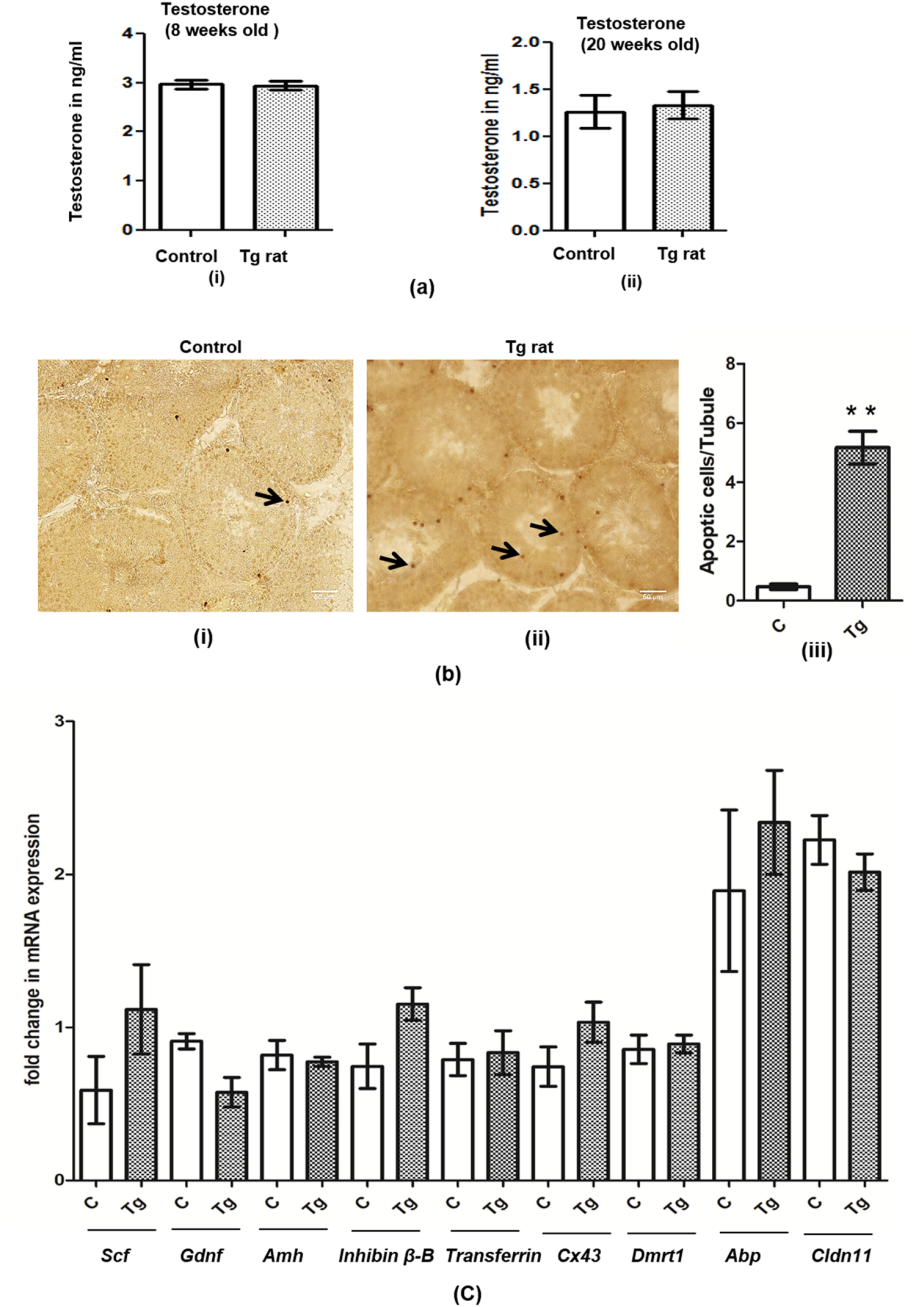
Effect of *Sostdc1* on testicular cells in the transgenic (Tg) rats. (**a**) *Sostdc1* did not affect the Leydig cell function in the Tg rat. (i) The level of testosterone (T) in ng/ml in the blood of Tg rats (8 weeks old rat) and age matched WT control rats were analyzed by RIA. *P = 0.05; significant difference in relation to normal littermates. n ≥ 7. (ii) The level of testosterone (T) in ng/ml in the blood of Tg rats (20 weeks old) and age matched control rats were analyzed by RIA. *P = 0.05; significant difference in relation to normal littermates. n ≥ 7. (**b**) Increase in germ cell apoptosis in the testis of transgenic rat. Tunnel assay to detect apoptosis was performed on the testis cross section of 20 weeks old control rat (i) and Tg rat (ii). Arrows showed the presence of apoptotic cells. Please note the increase in the number of apoptotic cell in the testis of Tg rat as compared to that of control (iii). All images were taken at a total magnification of 20X. *P ≤ 0.05. (**c**) *Sostdc1* did not affect the Sertoli cell maturational markers in the Tg rat. Q-RT-PCR of analysis showing relative expression levels of various Sc maturational status genes (*Scf*, *Gdnf*, *Amh*, *Inhibinβ-Β*, *Transferrin*, *Connexin* 43, *Dmrt1*, *Abp* and *Claudin* 11) in the testis of control and Tg rat (both of 20 weeks of age), n ≥ 3, one-way ANOVA followed by Dunnett’s post-test.

Testosterone (T) levels were measured in Tg rats of 8 weeks old and 20 weeks old to ascertain the effect of *Sostdc1* on adult Leydig cells (Lc) function. There were no significant (p ≤ 0.05) changes in the T levels of Tg rats as compared to that of the age-matched WT control rats both for 8 weeks and 20 weeks of age (Fig. 3a) suggesting that the Lc activity was not altered by Sc specific *Sostdc1* expression.

#### Germ cells (Gc)

To determine if apoptosis of Gc contributes to the low sperm production in Tg rats, TUNNEL assays were performed on testicular sections obtained from 20 weeks of age. The apoptotic cells defined by TUNNEL-positive nuclei were mostly found in the seminiferous tubules **(**Fig. 3bi,ii**)**. However, the frequency of TUNEL-positive cells was variable among the tubules, but overall there were more numbers of apoptotic Gc in the seminiferous tubules of Tg rats as compared to that of the WT age-matched controls **(**Fig. 3biii**)**.

#### Sertoli cells (Sc)

To determine the effect of *Sostdc1* on the maturation status of Sc, qRT-PCR analysis of some well-defined maturation markers of Sc like *Scf*, *Gdnf*, *Amh*, *Inhibin –β-B*, *Transferrin*, *Connexin* 43, *Dmrt1*, *Abp*, *and Claudin11* were performed with the whole testicular extracts of the Tg and WT age-matched control rat testes (both from 20 weeks of age). The expression levels of these markers were found to be uniform between Tg and age-matched WT control testes suggesting that the maturational status of Sc remained unaffected despite the long term expression of *Sostdc1* in these cells (Fig. 3c**)**. Furthermore, in order to investigate any impact of *Sostdc1* expression on proliferation of Sc, we further analyzed the Sc number by staining with Sox9 (marker of Sc), and observed that the expression of *Sostdc1* in Sc did not affect their numbers in adult Tg testis of 20 weeks age (Supplementary Fig. S5).

### *Sostdc1* activated the BMP signaling but not the Wnt signaling by upregulating the phospho smad1/5/8 in the transgenic rat testis

**Figure 4:**
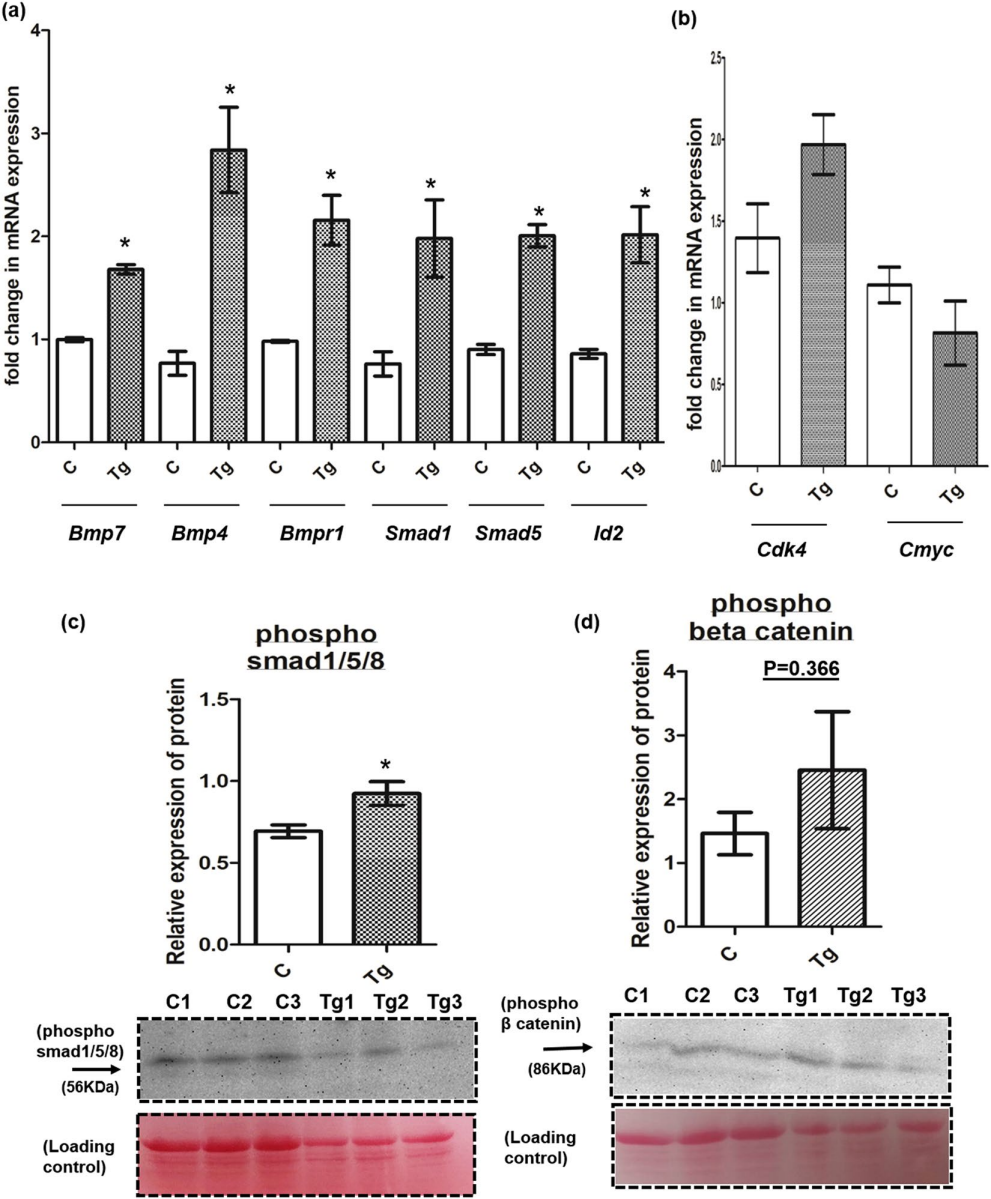
*Sostdc1* activated the BMP signaling but not the Wnt signalling in the transgenic (Tg) rat testes. (**a**) Q-RT-PCR of analysis of relative expression levels of BMP target genes in the testis of control rat and Tg rat. Level of BMP target genes (*Bmp7*, *Bmp4*, *Bmpr1*, *Smad1*, *Smad5*, *Id*_2_) were all significantly up regulated in the testis of Tg rat as compared to that of the age matched WT control (both of 20 weeks of age), n ≥ 3, *P = 0.05. (**b**) Q-RT-PCR of analysis of relative expression levels of Wnt target genes in the transgenic rat testes. Level of Wnt target genes (*Cdk4* and *Cmyc*) were all remained unchanged in the testes of transgenic rat as Tg rat as compared to that of the age matched WT control (both of 20 weeks of age), (n ≥ 3, *P ≤ 0.05). (**c**) The densitometric analysis of the western blot of the phospho smad 1/5/8 and (**d**) phospho β-catenin level in the testicular extract of three different transgenic (Tg1, Tg2, Tg3) and control (C1, C2, C3). The level of expression of phospho smad 1/5/8, a downstream transcription factor of BMP 2/4/7 signaling was significantly up regulated whereas the level of expression of phospho β- catenin, a downstream transcription factor of Wnt signaling was unaltered in the testis of Tg rat as compared to that of the age matched WT control (both of 20 weeks of age). Kindly note that, the normalization was performed with the whole protein by staining with Ponceau S in the same blot. Blots were cropped to fit into the figure. Full-length blots of phospho smad 1/5/8, phospho β-catenin and Ponceau S staining (for whole protein) were presented in Supplementary Figs S6 and S7, respectively. (n = 3, *P ≤ 0.05).

Q-RT-PCR analysis of the BMP regulated genes like *Bmp7*, *Bmp4*, *Smad1*, *Smad5*, *Bmpr1* and *Id*_2_ were performed from the whole testes of the Tg and control WT rats (both at 20 weeks of age) (Fig. 4a). All these genes were significantly (p ≤ 0.05) upregulated in the testes of Tg rats as compared to that of the age matched WT control rats. Interestingly, *Id*_2_, which induces apoptosis^36^, also was upregulated in the Tg rat testis as compared to that of the age-matched WT controls.

On the other hand, qRT-PCR analysis of the Wnt-regulated genes like *cmyc* and *cdk4* were also performed from the whole testes of the Tg and WT control rats (at both 20 weeks of age). However, there was no significant difference in the mRNA level of Wnt responsive *cmyc* and *cdk4* between Tg and control WT testes (Fig. 4b**)**.

To examine the mechanism involved in the up regulation of BMP responsive genes in Tg testis, we further performed the western blot for phospho smad 1/5/8 protein, a downstream transcription factor of BMP 2/4/7 signaling with the whole testicular protein extracts obtained from the Tg and WT testes (both at 20 weeks of age). We observed a significant (p ≤ 0.05) up regulation in the phospho smad 1/5/8 level in the Tg testes as compared to that of the age-matched WT controls **(**Fig. 4c**)**. These data suggested that *Sostdc1* mediated BMP signaling was up-regulated via activated phospho smad 1/5/8 level in the Tg testis. However, the level of phospho β-catenin protein, a downstream transcription factor of Wnt signaling was remained unchanged in the Tg testes as compared to that of WT control testes (Fig. 4d**)**.

### Phospho smad 1/5/8 levels were increased in the Gc of the transgenic rat testis

**Figure 5:**
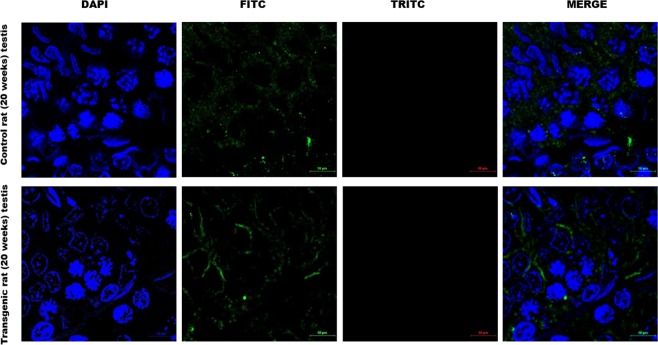
Localization of phospho smad 1/5/8 in male germ cell of adult rat testes. Immuno-histochemical localization of phospho smad 1/5/8 in testicular cross section of 20 weeks old WT control and age matched Tg rat. Localization of phospho smad 1/5/8 was more prominent in various stages of Gc of Tg testis. The image of secondary antibody control of pSmad 1/5/8 in the WT control testis (20 weeks old) was provided in Supplemental Fig. S8, Total Magnification, 300X.

To determine which cell types of adult (20 weeks of age) Tg testes contribute towards such increased levels of phospho smad 1/5/8, we further studied the localization of phospho smad 1/5/8 in testicular cross sections. We noticed the specific staining for phospho smad 1/5/8 in the various stages of Gc both in Tg rat testes and in the age matched WT controls (Fig. 5). However, no such staining were observed in the testis incubated with only secondary antibody (Supplementary Fig. S8**)**. These data suggested that the increase in phospho smad 1/5/8 levels in the Tg testes were mainly contributed by the Gc components.

### Expression of Sostdc1 was up-regulated in testis of infertile human male

The data from above study suggested that Sc specific *Sostdc1* expression during puberty leads to impaired spermatogenesis. We further analyzed the differential expression of *Sostdc1* in human testis sample using the microarray information of testis samples of infertile and control male from the available database (GSE 45887)^37^. This data showed that *Sostdc1* was significantly (p ≤ 0.05) upregulated (6.7 folds) in testis of infertile patients as compared to that of the control (Supplementary Fig. S9).

## Discussion

In this study, we have demonstrated that the persistent expression of *Sostdc1* in adult Sc leads to low sperm count during adulthood indicating that the pubertal down-regulation of *Sostdc1* in Sc plays a critical role in regulating male fertility. We concluded that *Sostdc1* is an essential negative regulator of spermatogenesis based on the following experimental evidences; firstly, *Sostdc1* expression was predominant in immature Sc as compared to that of the maturing Sc. Secondly, we provided strong *in vivo* evidence to establish that preventing the pubertal down-regulation of *Sostdc1*, specifically in Sc leads to impaired spermatogenic onset. Finally, the long term expression of *Sostdc1* in adult Sc, upregulated BMP signaling via phospho smad 1/5/8 pathway in the Tg testes resulting into Gc apoptosis.

*Sostdc1* is a dual Wnt/BMP regulator^15–18^. Its cellular localization has distinct effects on the Wnt/BMP pathway. It is reported that the loss of *Sostdc1* do not affect the Wnt responsive genes in mice^31^ which is consistent with our current data. However, a previous study suggests that loss of *Sostdc1* exhibit a significant decrease in the BMP responsive genes in mice^31^, supporting our present observation of upregulation of the BMP signaling due to increase in the level of *Sostdc1* in the in Tg rat testis. The intracellular *Sostdc1* is known to inhibit BMP signaling by binding to intracellular *BMP7* leading to their proteasomal degradation^38^. During puberty, various BMPs like *BMP7* are produced by the Gc but not the Sc^19^, which precludes the possibility of any intracellular inhibition of BMPs by the Sc specific *Sostdc1*.

Our data suggested that the augmentation of BMP signaling were associated with the increased Gc apoptosis in the Tg rat testis. This is supported by previous report where the upregulation of BMP signaling in CIZ−/− knockout mice induce apoptosis of Gc causing infertility^39^. Moreover, the ectopic expression of *Bmp* 2*/4/7* are known to trigger apoptosis during limb development^40–42^. *Bmp7* has also been shown to upregulate Inhibitor of Differentiation 2 (*Id*_2_)^43^. We here have also observed a significant upregulation of both *Bmp7* and *Id*_2_ in the Tg testis, probably preventing differentiation of Gc. *Id*_2_ is known to promote apoptosis in cell by a novel mechanism independent of dimerization to basic helix-loop-helix factors^36^. Therefore, it is reasonable to consider that the *Sostdc1* mediated augmentation of BMP signaling might have induced Gc apoptosis in the Tg rats.

A shift in the localization of phospho smad 1/5/8 from Sc to Gc during pubertal development has already been reported^19^. Previous studies also showed that the upregulated expression of phospho smad 1/5/8 level leads to apoptosis^44^. Furthermore, phospho smad1/5/8 signaling is known to be detected in the testis of infertile man, and it is more prominent in Gc indicating that phospho smad1/5/8 signaling may have a role in male infertility^45^. In this context, using data from a resource study (GSE 45887)^37^, we further observed the expression of *Sostdc1* was upregulated in the testis of infertile patients. Therefore, these two different observations from independent sources collectively established a probable correlation between high *Sostdc1* expression and activated phospho smad 1/5/8 signaling with human male infertility, supporting our present findings in Tg rats.

In summary, the present study provided a new insight on Sostdc1 mediated regulation of male fertility. Our data indicated that Sc specific persistent expression of *Sostdc1* leads to progressive Gc apoptosis via the dysregulation of the BMP signaling. Additionally, this study further established the importance of *Sostdc1* in unresolved cases of certain forms of idiopathic male infertility where failure of the natural down-regulation of *Sosdc1* during puberty, may be one of the reason of insufficient sperm production. In 25–30% of men with spermatogenic failure, causes of infertility are unknown, and they are not amenable to cure with any of the conventional hormonal therapy^5–7^. Therefore, delineating the cellular causes of male infertility by such functional genomics studies may be helpful in determining non-hormonal factors which may be assessed to evaluate basis of idiopathic male infertility.

## Materials and Methods

### Animals and reagents

Wistar rats (*Rattus norvegicus*) were obtained from the Small Animal Facility of the National Institute of Immunology (New Delhi, India). All animals were housed and used as per the national guidelines provided by the Committee for the Purpose of Control and Supervision of Experiments on Animals. Protocols for the experiments were approved by the Institutional Animal Ethics Committee (IAEC) of National Institute of Immunology (New Delhi, India) and the Committee for the Purpose of Control and Supervision of Experiments on Animals (CPCSEA). All other reagents, unless stated otherwise, were procured from Sigma Chemical (St. Louis, MO).

### Differential expression analysis of *Sostdc1* in 5 days and 12 days old rat Sc culture

The differential expression of *Sostdc1* was obtained from our previously reported microarray analysis (GSE48795)^14^ using 5 days and 12 days old rat Sc^12–14^. The differential expression of *Sostdc1* from the microarray analysis was validated by q-RT-PCR analysis in three separate sets of Sc cultures of both the age groups. For this, Sc were isolated and cultured from 5 days and 12 days old rat testis as reported by us previously^11^. These cells were treated with hormones^46^ (FSH and Testosterone in combination) on day 4 of culture similar to the treatment given to cells for microarray analysis^14^. Total RNAs were extracted from these Sc as described previously^11^. Testes from about 20–30, and 10–20 male rats were pooled for 5 days and 12 days old rat Sc cultures, respectively.

### Generation of transgenic (Tg) rats expressing *Sostdc1* in Sc

Annotated cDNA clones coding for murine *Sostdc1* was procured from IMAGE cDNA consortium through open biosystems (Thermo Scientific co., USA). A flag tag sequence (DYKDDDDK) was added to the c terminus of the ORF of *Sostdc1* by PCR, and were cloned downstream of the Rhox5 promoter in an IRES 2 EGFP vector backbone. Primer details were given in Supplementary Table S1. The construct was linearized with *Stu I*, and was used to generate transgenic rat (Tg) as reported by us previously^47^. Briefly, the linearized construct (a total of 30 µg DNA with a concentration of 1 µg/µl) was injected into the testis of 38 days old Wistar rat, and electroporated using 8 square 90 V electric pulses in alternating direction with a time constant of 0.05 second and an inter-pulse interval of ~1 second via an electric pulse generator. The electroporated male rats were mated with wild-type female rats 60 days post electroporation. Born pups were screened by PCR using genomic DNA obtained from their tail biopsies and those positive for the transgene, were regarded as F_1_ generation of transgenic (Tg) animals. Primer details were given in Supplementary Table S1. The southern blot analysis was performed for propagation of transgene using the probe for GFP as described by us previously^47^. Briefly, the *EcoRV* digested DNA was resolved on a 1% agarose gel followed by denaturation and neutralization. DNA was transferred to nylon membrane (Hybond N+, Amersham Pharmacia Biotech) using 10X sodium citrate buffer for overnight. Probe was prepared by amplifying a fragment that contained EGFP gene (633 bp) using the P1: GACGTAAACGGCCACAAGTT, P2: GGCGGTCACGAACTCCAG primers by PCR and was labeled with DIG-11-UTP using High Prime DNA labeling kit (Roche Diagnostic GmbH, Mannheim, Germany) during PCR amplification. The membranes were pre-hybridized for 1 hour at 45 °C in hybrisol (EMD Millipore, a division of Merck KGaA, Darmstadt, Germany) without labelled probe and then hybridized separately at 60 °C with specific DNA probes for 12 hour. The membranes were washed twice for 5 min each, at room temperature in 2Х saline sodium citrate buffer and 0.1% SDS. This was followed by another washing for 15 min at 60 °C (in 0.5Х saline sodium citrate buffer and 0.1% SDS). Detection was performed by exposing to X ray film.

### Semi-quantitative RT-PCR for transgene expression

The Tg and age matched wild type (WT) control rats were sacrificed, and various organs (liver, spleen, kidney, epididymis and testis) were collected. They were snap-frozen in liquid nitrogen and stored in −70 °C until used. Later, the organs were removed from freezer, weighed and were then homogenized/grind thoroughly in a DEPC-treated RNase-free pestle mortar (pre-cooled with liquid nitrogen) in presence of liquid nitrogen and transferred to sterile RNase-free microfuge tubes. RNA was isolated and PCR was performed with specific primer for the transgene (*Sostdc1 IRES EGFP*), described in the Supplementary Table -S1. *Ppia* was used as internal control.

### Western blot analysis

The Tg and age matched WT rats were sacrificed and various organs (liver, spleen, kidney, epididymis and testis) were collected for protein isolation. For flag-tagged recombinant protein detection, blot was incubated for overnight at 4 °C with rabbit antiserum against flag (Abcam, Ab- 21536) at a dilution of 1:5000. The secondary IgG antibody was used at a dilution of 1:8000 for 1 hour at room temperature. Probe for β- actin was used as endogenous control in the same blot. For detection of phospho smad protein and phospho β-catenin protein in the testicular lysate, goat-phospo Smad 1/5/8 antibody (sc-12353) and rabbit- phospho β-catenin antibody (orb 5769) were used at a dilution of 1:1000 at 4 °C for overnight. The secondary antibody used for phospho smad 1/5/8 was anti goat HRP (Invitrogen, USA -611628) at a dilution of 1:3000 and for phospho β-catenin the secondary antibody was goat anti-rabbit HRP (Thermoscientific-353-1) at a dilution of 1:3000 at room temperature for 1 hour. The loading control for whole protein was performed in the same blot by staining with Ponceau S and was used for normalization. The densitometry was performed with α-imager.

### Immunohistochemistry (IHC)

The testis of Tg and age matched WT rat were collected, and fixed in Bouins solution, and IHC were performed as described by us before^47^. To stain for transgene flag-tagged protein, primary antibody used was Rabbit polyclonal anti-Flag antibody (Abcam, Ab- 21536) at a dilution of 1:250 for 2 hour at room temperature and the secondary antibody was goat anti-rabbit IgG conjugated with HRP (Thermoscientific, USA) at a dilution 1:1000. For localization of phospho smad 1/5/8 protein in the cross-section of testis, goat polyclonal anti phospho smad 1/5/8 (SC-12353) was used at a dilution of 1:1000 for 2 hour at room temperature, and anti-goat-Alexa488 was used at a concentration of 1:3000 for 1 hour at room temperature. To stain for Sox9 (marker of Sc), primary antibody used was Rabbit polyclonal anti-Sox9 antibody (Abcam, Ab- 26414) at a dilution of 1:1000 for 2 hour at room temperature and the secondary antibody was goat anti-rabbit IgG conjugated with Alexa488 (Thermo scientific, USA) at a dilution 1:3000. The number of Sox9-positive cells per total number of seminiferous tubules within each testis cross-section was counted to determine the number of Sc per tubule. A total of three cross sections per animal was analyzed for determining the number of Sc per tubule at a total magnification of 20X.

### Fertility analyses of *Sostdc1* expressing Tg rats

Testes of both Tg and WT rats were removed at the age of 8 weeks and 20 weeks. Total sperm present in both epididymis of each rat was counted after releasing the sperm in 3 ml of 1X PBS by puncturing epididymis at several sites and squeezing. One Tg male from each F_1_ and F_2_ generations was cohabitated with two healthy WT females for three months. This ensured exposure of female to the male at least through five ovarian cycles per month. To determine the libido of the F_1_ and F_2_ Tg male rats, copulatory vaginal plugs were investigated 12 hour after mating for each pair. Litter size was determined after delivery of progeny to assess fertility of parents. Similarly, WT rats were also assessed for fertility.

### Tissue histology

Tissue histology was performed as described by us previously^48^. Briefly, after castration, testicular tissue samples of rats were fixed in Bouins solution at room temperature for 24 hour. Dehydration of tissues was done in a series of ascending concentrations of ethanol for 1 hour in each grade of ethanol. The tissues were embedded in paraffin, and 4 µm sections were cut. Sections were stained with hematoxylin and eosin, and were examined for evaluating the status of spermatogenesis.

### Serum T RIA

Blood was obtained through retro-orbital bleeding before sacrificing the rats following all the ethical measures. Serum testosterone (T) levels of rats were measured as described by us^48^. Serum T levels from the Tg and WT rats were assessed at 8 weeks and 20 weeks of age. Serum T levels was assayed by RIA in triplicate. The intra- and inter assay coefficients of variation were less than 7% and less than 10%, respectively.

### Tunnel assay

Testes were collected from both Tg and WT rats at the age of 20 weeks, and were fixed in Bouins solution. Testicular paraffin sections were deparaffinized, rehydrated by successive serial washings with ethanol and treated with proteinase K for permeabilization of cells. Fragmented DNA was labeled with terminal deoxynucleotidyl transferase and biotin dNTPs. The streptavidin-horseradish peroxidase and diaminobenzidine tetra hydrochloride system was used to visualize for the apoptotic cells under light microscope. The number of TUNEL-positive cells per total number of seminiferous tubules within each testis cross-section was counted to determine the incidence of apoptosis in the WT and Tg testes as described previously^49–51^. A total of three cross sections per animal was analyzed for TUNEL assay at a total magnification of 20X.

### Q-RT-PCR for gene expression studies in Tg testes

Total RNA was extracted from the whole testicular extract of control and Tg rats. q-RT-PCR was performed to detect expression of *Sostdc1* and different targets of BMP like *Bmp4*, *Bmp7*, *Bmpr1*, *Smad1*, *Smad5*, *Id*_*2*_ and Wnt target genes such as *Cmyc*, *Cdk4*, various Sc maturation markers like *Scf*, *Gdnf*, *Amh*, *Inhibin βΒ*, *Transferrin*, *Dmrt1*, *ABP*, *Connexin* 43 and *Claudin* 11. q-RT-PCR amplifications were performed using the RealplexS (Eppendorf) as described by us before^11^. The expressions of mRNA of the target genes were evaluated by the efficiency corrected 2^−ΔΔCT^ method. The list of genes with corresponding primer sequences are given in the Supplementary Table S1.

### Differential expression analysis of *Sostdc1* in infertile human testis

The microarray data of testis samples of infertile and age-matched control human male was obtained from the public data base - GSE45887^37^. In all of these patients, the well -known causes of infertility such as Y-chromosome microdeletions, cystic fibrosis transmembrane conductance regulator mutation, antisperm antibodies (ASA), orchitis, testicular torsion, and varicocele have been excluded. Patients fell into different categories based on histopathologic images. The difference in expression of *Sostdc1* in fold change was expressed in log 2 scale between control (n = 2) and infertile patients having post meiotic arrest and meiotic arrest (n = 5).

### Statistical analysis

For the validation of differential expression of *Sostdc1* by microarray analysis, at least three sets of independent cultures conducted on three different calendar dates, for each age group (5 days and 12 days old rats) were used to interpret the data using paired *t*- test. Similar experiments were performed for analyzing gene expression by q-RT-PCR analysis and data was analyzed using Mann–Whitney test. For fertility or gene and protein expression studies, each set of observation was pooled from at least 3 or more individual rats (both WT and Tg) and at least 3 sets of observations were analyzed and represented by Mann–Whitney test as a data. The differential expression of *Sostdc1* in the infertile patient sample and control male were analyzed using Mann–Whitney test. A value of P ≤ 0.05 was considered as significant. All statistical analyses were performed using GraphPad Prism v. 5.01,(GraphPad Software, Lajolla, CA, USA).

## Supplementary information

Supplementary Info

## Data Availability

No datasets were generated or analyzed during the current study.
