# Supplementary material for: Downregulation of Sostdc1 in Testicular Sertoli Cells is Prerequisite for Onset of Robust Spermatogenesis at Puberty
Source: Sci Rep. 2019 Aug 7;9:11458. doi: 10.1038/s41598-019-47930-x (PMC6686024; doi:10.1038/s41598-019-47930-x)
Supplement: Supplementary file 1 — Supplementary Info [file 41598_2019_47930_MOESM1_ESM.doc]

**Supplemental Information**

**Downregulation of Sostdc1 in Testicular Sertoli Cells is Prerequisite for Onset of Robust Spermatogenesis at Puberty**

Bhola Shankar Pradhan^1^, Indrashis Bhattacharya^1,2^ Rajesh Sarkar^1^ and Subeer S Majumdar^1,3*^

^1^Cellular Endocrinology Laboratory, National Institute of Immunology, Aruna Asaf Ali Marg, JNU complex, New Delhi 110067, India.

^2^Present Address Department of Zoology and Biotechnology, HNB Garhwal University, Srinagar-246174, Uttarakhand, India

^3^National Institute of Animal Biotechnology, Hyderabad, Telangana, India-500 049

**Keywords : Spermatogenesis; transgenic rat; *Sostdc1*; Sertoli cells; male infertility**

*Corresponding author:

Subeer S. Majumdar, Cellular Endocrinology Laboratory, National Institute of Immunology, ArunaAsaf Ali Marg, JNU complex, New Delhi 110067, India, Telephone: +91-11-26703751, Email : [subeer@nii.ac.in](mailto:subeer@nii.ac.in)


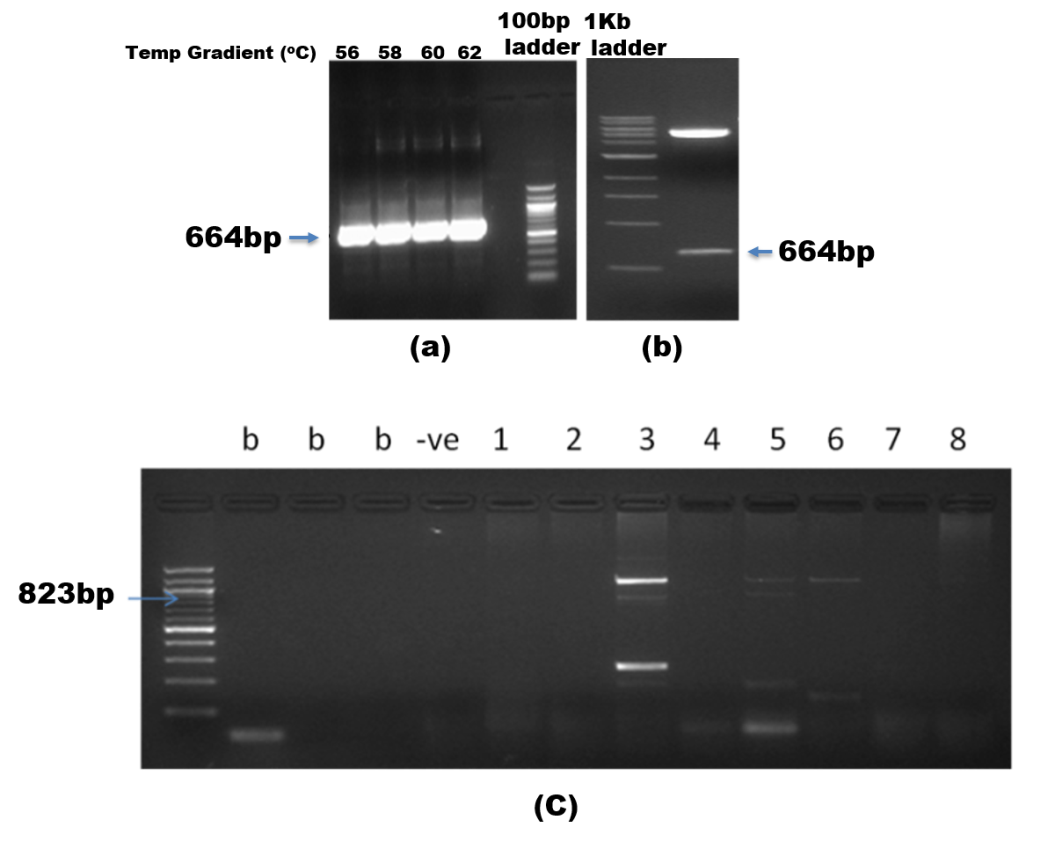


**Supplemental Figure S1 Cloning of *Sostdc1* under proximal Rhox5 promoter and PCR screening of transgenic (Tg) rat.**

**(a)** Amplification of the full length ORF of *Sostdc1* by PCR. The PCR was performed with 4mM MgCL_2_ concentration at different temperature (56^o^C, 58^o^C, 60^o^C and 62^o^C), and an amplicon of 664 bp was the desired product (full length ORF of *Sostdc1* with flag tag sequence*)*. **(b)** The *Sostdc1* with the flag tag sequence (DYKDDDDK) was cloned downstream of proximal Rhox5 promoter between the enzyme sites *Xho I* and *Sac II.* The fragment size of 664 bp was the desired product (full length ORF of *Sostdc1* with flag tag sequence*)*. **(c)** Genotyping of transgenic rats. The genotyping was performed by PCR from the tail DNA of the progenies of *Sostdc1* overexpression fore founder with the transgene specific primers. b refers to blank i.e. the PCR master mix without any genomic DNA. 1-8 refers to the progenies of *Sostdc1* over-expression fore founder. -ve refers to wild type (WT) control rat. Amplicon of 823 bps confirmed the presence of the transgene. **
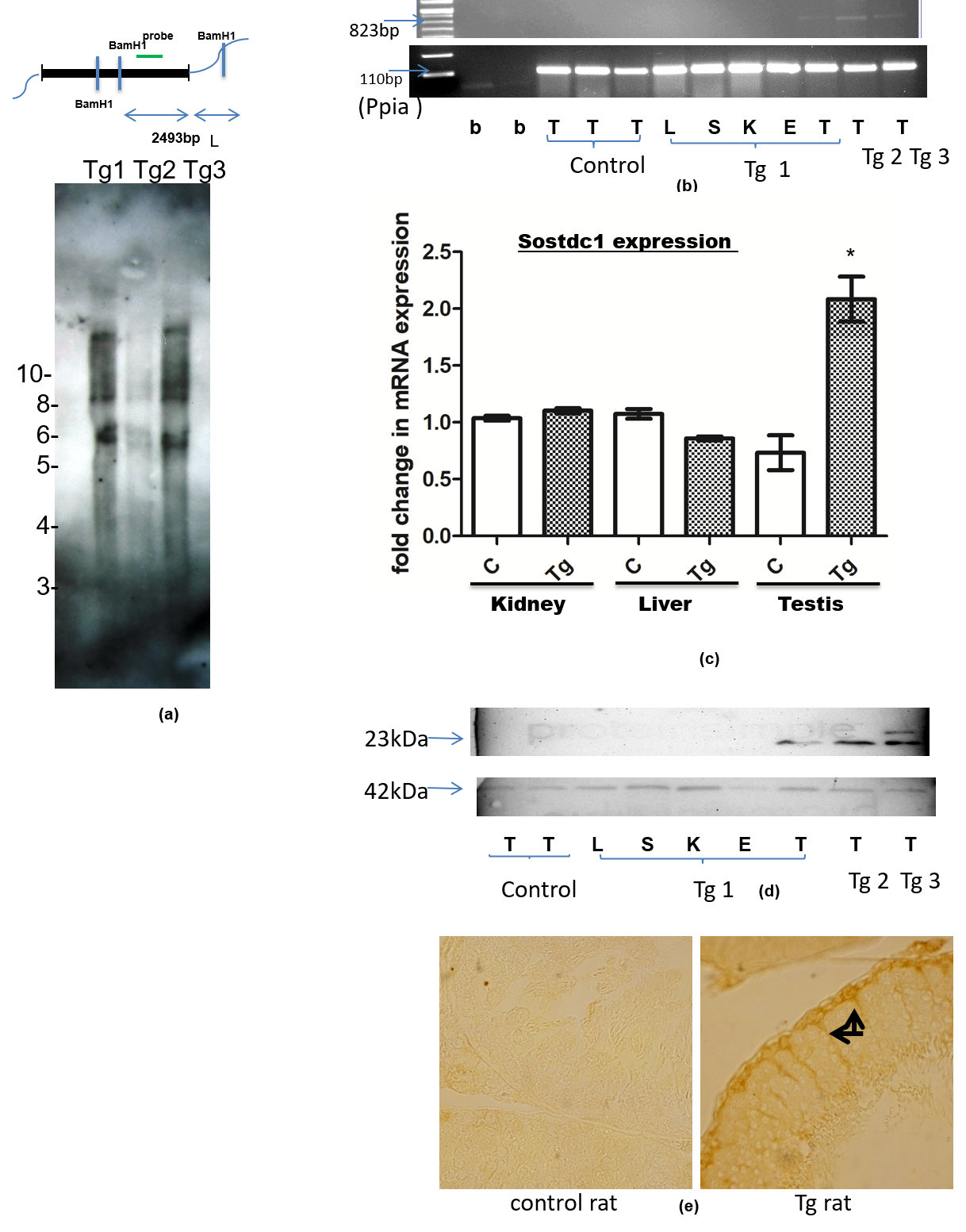
**

**Supplemental Figure S2. Generation of transgenic (Tg) rats expressing *Sostdc1* in Sertoli cells.**

**(a) Southern blot analysis of *Sostdc1* over expressing transgenic rat.** Genomic DNA of the transgenic rats were digested with *BamH1* and hybridized with GFP probe. Lane 1, 2, 3 represented the transgenic rat from three different lines. A band size of above 2493 bp was expected in the transgenic rat lines. Analysis of lanes 1-3 revealed for the presence of multiple copies of insertions.

**(b) Validation of the expression of the integrated transgene by RT-PCR.** Evaluation for the presence of *Sostdc1* IRES GFP transcript in the total mRNA of liver (L), spleen (S), kidney (K), epididymis (E), testes (T) of transgenic rats (Tg animal numbers were coded by 1,2,3) and testes (T) of wild type (WT) control (C) rats by RT-PCR, b stands for blank. Ppia was used as the internal control. The transcript (823 bp) specific for the transgene were detected only in the testes of transgenic rats ( Tg Number 1, 2 and 3).

**(c) Validation of the expression of the integrated transgene by qRT-PCR.** *Sostdc1* was significantly (p < 0.05) up regulated in the testicular extracts of transgenic (Tg) rats as compared to other organs at 20 weeks of age. However, no such rise was observed in the expression of *Sostdc1* mRNA in age matched wild type (WT) control (C) rats.

**(d) Validation of the expression of the integrated transgene by western blot analysis.** Western blot of protein extracted from various organs e.g. liver (L), spleen (S), kidney (K), epididymis (E) and testis (T) obtained from 20 weeks old transgenic (Tg) rats and wild type (WT) control (C) rats for detecting the *Sostdc1*-flag protein by flag specific antibody. 23 kDa specific protein was expressed only in testes of transgenic rats (Tg Number 1, 2 and 3). β-actin was used as loading control.

**(e) Validation of the expression of the integrated transgene by immunohistochemistry.** Immunohistohemistry was performed on the testicular sections of 20 weeks old transgenic (Tg) rats and that of the age matched wild type (WT) control rats for detecting *Sostdc1*-flag fusion protein by flag specific antibody. Specific staining was observed in only the Sc of the transgenic rat testes, whereas no staining was detected in the control. Black arrows represent the Sertoli cells in the testis. Total magnification, 60 X.


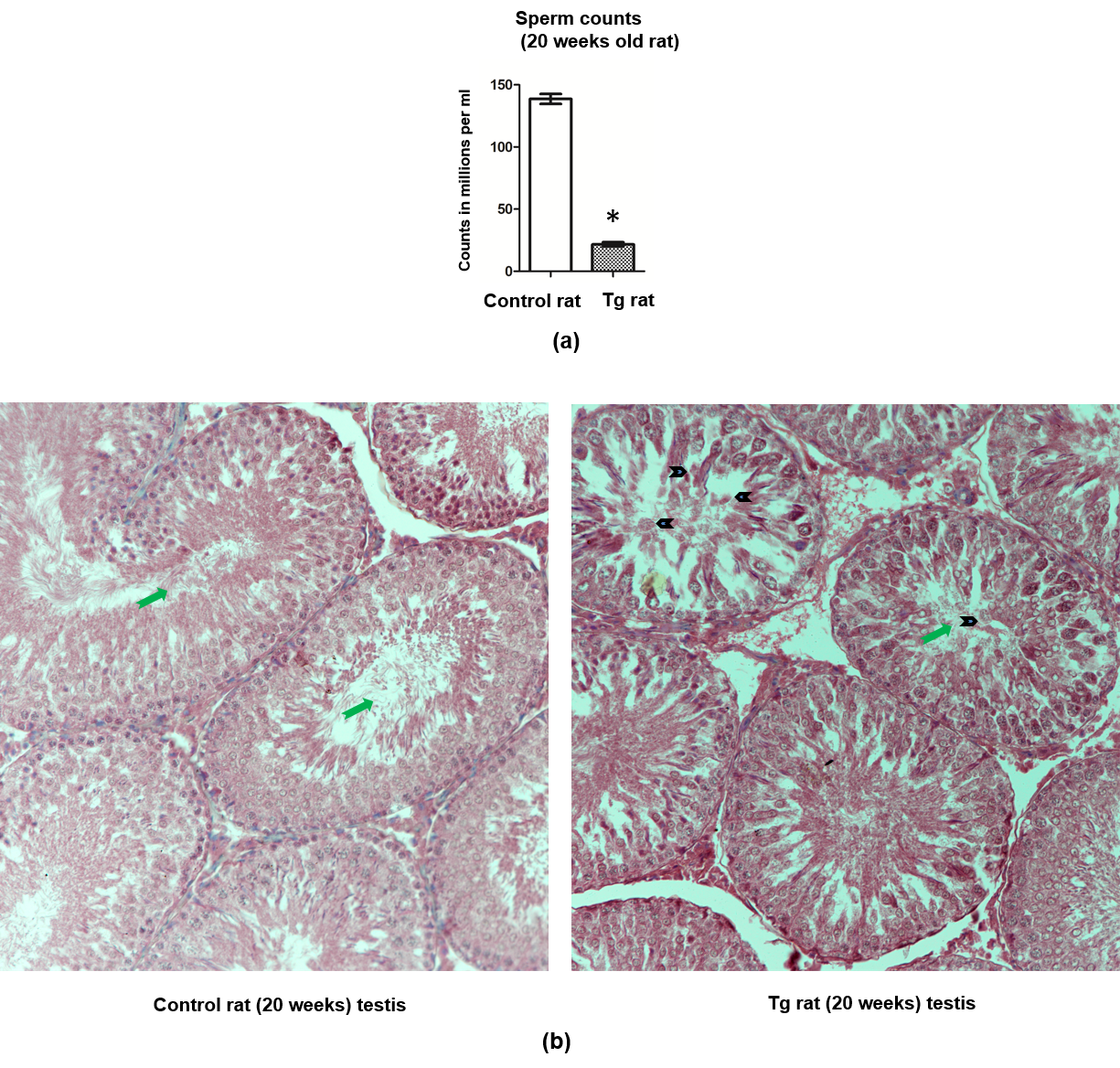


**Supplemental Figure S3.** **Spermatogenesis was impaired in the transgenic (Tg) rats (F_1_ generation) expressing *Sostdc1* in Sertoli cells post-pubertally.**

**(a)** The caudal epididymal sperm counts in the Tg rats (20 weeks old from F_1_ generation) and that of the age matched wild type (WT) control rats, *P ≤ 0.05; significant difference in relation to control. n≥3.

**(b)** Haematoxylin - eosin staining of testicular sections of Tg rats (20 weeks old from F_1_ generation) demonstrating low sperm density (in green arrows) in the lumen as compared to that of the age matched wild type (WT) control rat testis, the sloughing up of Germ cells (in black arrow heads) were predominantly observed only in the Tg rat testis. All images were taken at a total magnification of 20X.

**
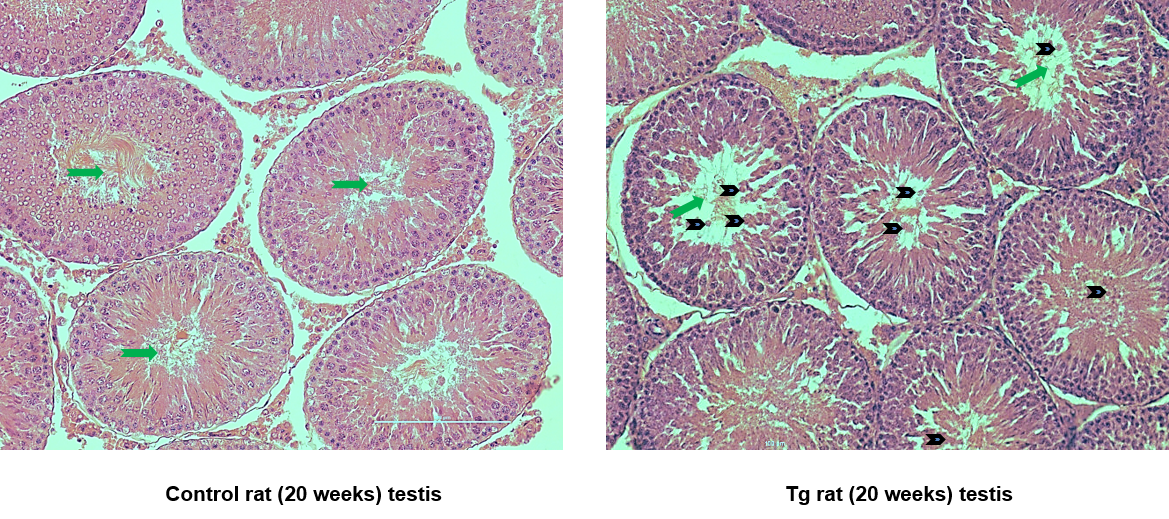
**

**Supplemental Figure S4.** **Spermatogenesis was impaired in the transgenic (Tg) rats (F_2_ generation) expressing *Sostdc1* in Sertoli cells post-pubertally.**

Haematoxylin-eosin staining of testicular sections of Tg rats (20 weeks old F_2_ generation) demonstrating low sperm density (green arrows) in the lumen as compared to that of the age matched wild type (WT) control rat testis and the sloughing up of Germ cells (black arrow heads) were predominantly observed only in the Tg rat testis. All images were taken at a total magnification of 20X.


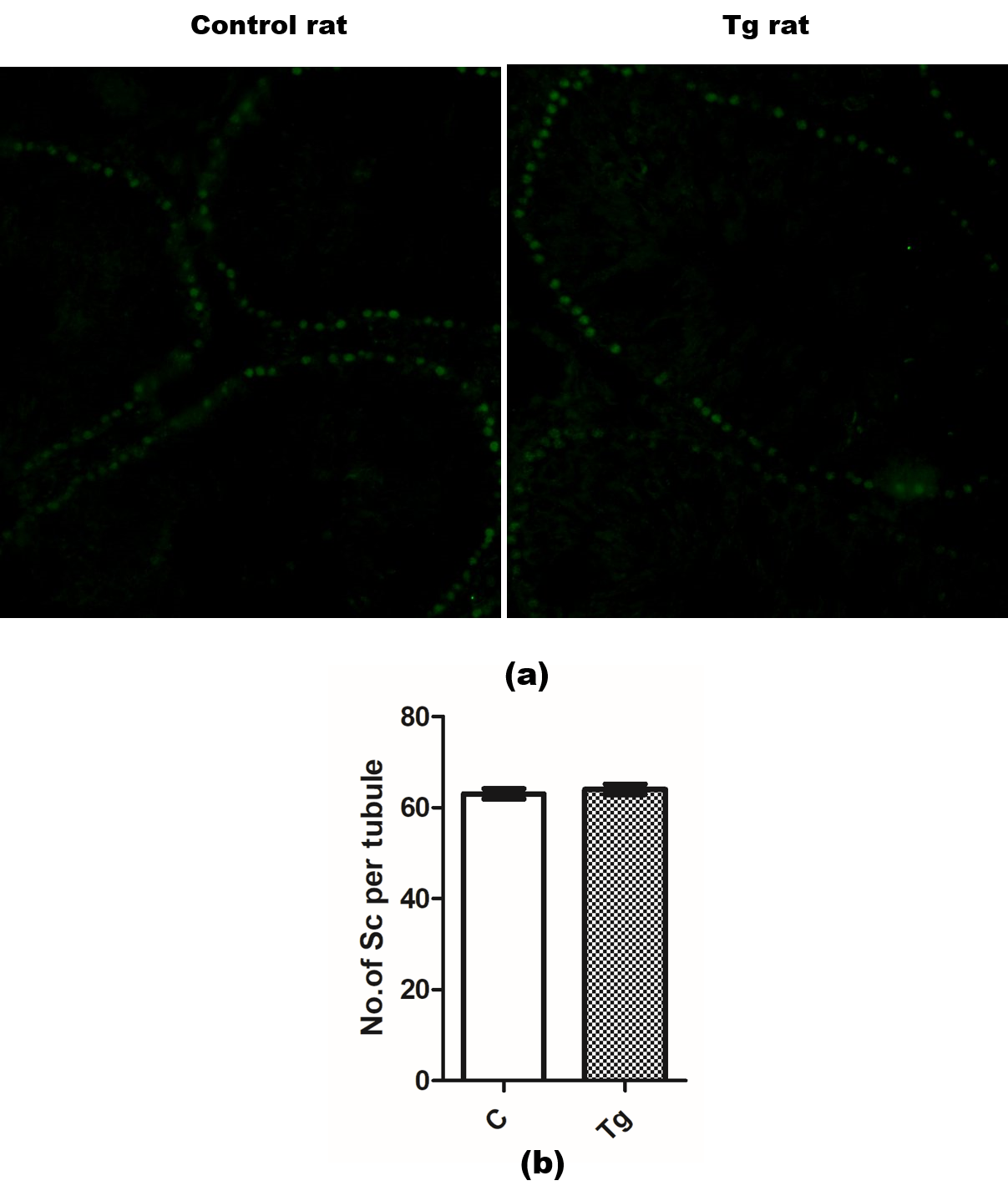


**Supplemental Figure S5. *Sostdc1* expression did not affect the number of Sertoli cells in the testes of transgenic (Tg) rats.**

**(a)** Immunohistohemistry was performed on the testicular sections of 20 weeks old *Sostdc1* over-expressing transgenic (Tg) rats and that of the age matched wild type (WT) control (C) rats for *Sox9* protein, a specific marker for Sertoli cell. Specific staining was observed in the Sertoli cells of both the control (C) and transgenic (Tg) rat testis.

**(b)** There were no significant difference observed in the total number of Sertoli cells per tubule in the testes of *Sostdc1* over-expressing transgenic (Tg) rats as compared to that of the age matched wild type (WT) control (C) rats. Total magnification, 20 X.

**
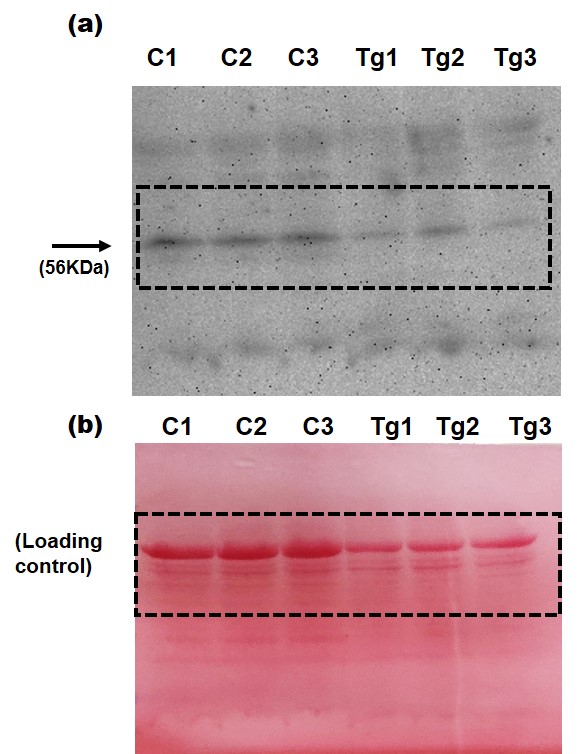
**

**Supplemental Figure S6. Full blot of phospho Smad 1/5/8 staining.**

**(a)** The western blot detecting the phospho Smad 1/5/8 levels in the testicular extracts of three different transgenic (Tg) rats (Tg1, Tg2 , Tg3) and age matched wild type (WT) Control rats (C1, C2,C3), all 20 weeks old.

**(b)** The loading controls for whole protein were also performed in the same blot by staining with Ponceau S and used for normalization.

**
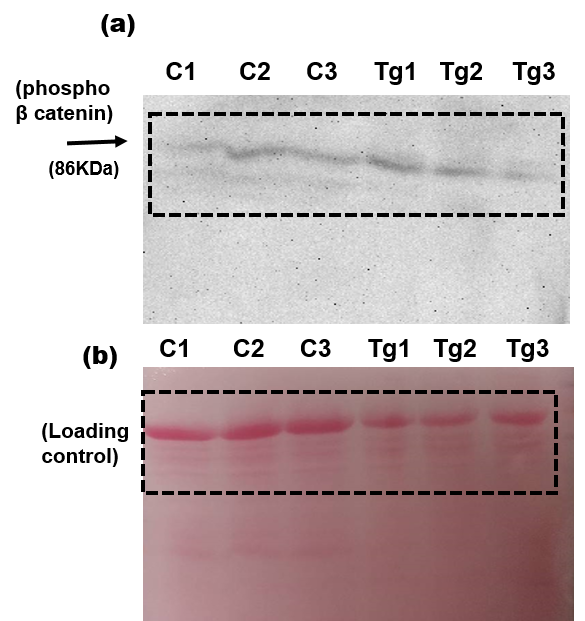
**

**Supplemental Figure S7. Full blot of phospho β- catenin staining.**

**(a)** The western blot detecting the phospho β-catenin levels in the testicular extracts of three different transgenic (Tg) rats (Tg1, Tg2 , Tg3) and age matched wild type (WT) Control rats (C1, C2,C3), all 20 weeks old.

**(b)** The loading controls for the whole protein were performed in the same blot by staining with Ponceau S and used for normalization.


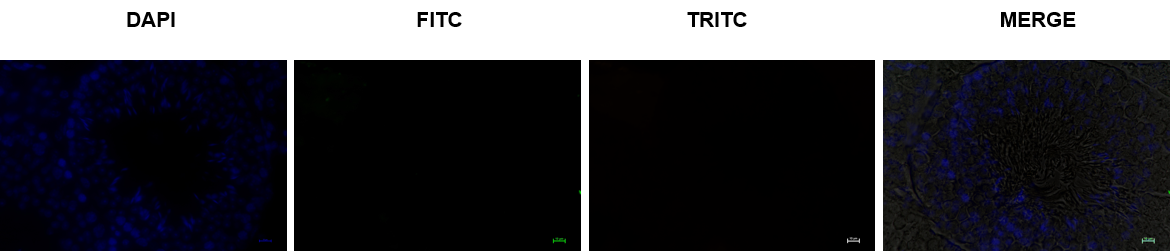


**Supplemental Figure S8.** **Secondary antibody control of phoshpo Smad 1/5/8 staining.** The testicular sections of 20 weeks old wild type (WT) rats were incubated only with anti-goat Alexa 488 to analyze the specificity of antibody against phospho Smad 1/5/8. All images were taken at a total magnification of 60X.


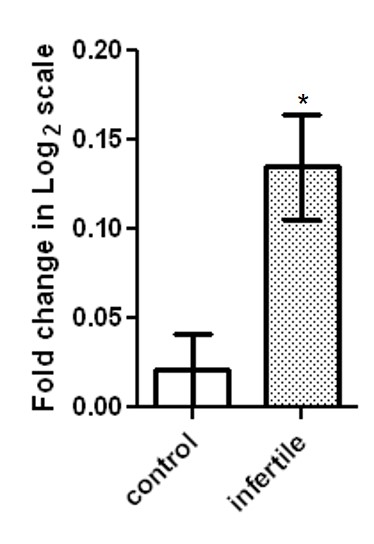


**Supplemental Figure S9. Differential expression of *Sostdc1* mRNA in testis of infertile human patients.**

*Sostdc1* mRNA expression was analyzed from the microarray data obtained from infertile male patients (n=5) and healthy control men (n=2). The fold change was expressed in Log_2_ scale, unpaired *t- test* followed by Welch correction was performed, * P ≤0.05.

**Supplementary Table S1.** Primers details used in the study.

| **Gene name** | **Sequence** | **Gene ID** | **Tm** | **Product size (bp)** |
| --- | --- | --- | --- | --- |
| Sostdc1(F) | AGTGCTTCCCAACTGGATCG | 266803 | 60 | 186 |
| Sostdc1(R) | CGGGTGTACCTCTTGCACTT | | 60 | 186 |
| Sostdc1 (F)  (Cloning) | TAACATCTCGAGGCCACCATGCTTCCTCCTGCCATTCA | | 60 | 664 |
| Sostdc1(R)  (Cloning) | TAACTGCCGCGGTTACTTGTCATCGTCATCCTTGTAATCGCTCAGACTGTGCTT | | 60 | 664 |
| Sostdc1(F)  (Screening) | ACGTCTGTAGCGACCCTTTG | | 60 | 823 |
| IRES (R) (Screening) | TTCTTCTGCTTGTCGGCCAT | | 60 | 823 |
| BMP 7 (F) | CGTCCAGACACTGGTTCACT | 85272 | 60 | 118 |
| BMP 7(R) | CAGGATGACGTTGGAGCTGT | | 60 | 118 |
| BMP 4(F) | ACTTCGAGGCGACACTTCTG | 25296 | 60 | 219 |
| BMP 4(R) | TCTCCAGATGTTCTTCGTGATGG | | 60 | 219 |
| SMAD1(F) | CGACACATCGGGAAAGGAGTC | 25671 | 60 | 209 |
| SMAD1(R) | TTCACAGACTGCGCCAGTAG | | 60 | 209 |
| SMAD5(F) | TGTTGGGCTGGAAACAAGGT | 59328 | 60 | 148 |
| SMAD5(R) | GTGACACACTTGCTTGGCTG | | 60 | 148 |
| BMPR1(F) | GATGGTTCGGCAGGTTGGTA | 81507 | 60 | 148 |
| BMPR1(R) | ACGCATTAACACCGTCTGGT | | 60 | 148 |
| ID2(F) | CTTGCAGGCGTCTGAATTCCC | 25587 | 60 | 277 |
| ID2(R) | GGAGACACCTGGGGAGATGA | | 60 | 277 |
| cMYC(F) | GGGAAAAAGAAGCGAGGGGAG | 24577 | 60 | 191 |
| cMYC(R) | CAAAGCCCTTCTCACTCCAGA | | 60 | 191 |
| CDK4(F) | GATGCGCCAGTTTCTAAGCG | 94201 | 60 | 153 |
| CDK4(R) | AGGGCCATCTGGTAGCTGTA | | 60 | 153 |
| SCF(F) | GTGGATGACCTCGTGGCATGTA | 60427 | 60 | 155 |
| SCF(R) | TCAGATGCCACCATGAAGTCC | | 60 | 155 |
| GDNF(F) | GGCCGACAATGTACGAC | 25453 | 60 | 172 |
| GDNF(R) | CCACACCGTTTAGCGGAA | | 60 | 172 |
| Claudin  11(F) | ACGGTTGCGTATGCTTTGA | 84588 | 60 | 131 |
| Claudin  11(R) | ACACCCATGAAGCCAAATT | | 60 | 131 |
| Transferrin(F) | TCTGTTTGTTCCGGTCTTCC | 24825 | 60 | 203 |
| Transferrin(R) | GCACCCACCTCTTGGATTT | | 60 | 203 |
| Inhibinβ(F) | TCCTAGTGCCCTGCTGAGAT | 25196 | 60 | 158 |
| Inhibinβ(R) | ACCCACAGGGACAACTTCTG | | 60 | 158 |
| MIS(F) | GGAGACCTACCAAGCCAACA | 25378 | 60 | 186 |
| MIS(R) | TCCTCCGACAGGCTGATG | | 60 | 186 |
| Connexin 43(F) | GTCTACCCCTCTGGGTGTGA | 24392 | 60 | 180 |
| Connexin 43(R) | AGGACCAGTCGAGGATGATG | | 60 | 180 |
| Dmrt1(F) | GGTCAGAGCATGTCCCAGAT | 114498 | 60 | 182 |
| Dmrt1(R) | GGTTCAGAGGACGCAGACTC | | 60 | 182 |
| ABP (Shbg) (F) | AGGGTTTGCTGATTTTGGTG | 24775 | 60 | 129 |
| ABP (Shbg) (R) | GACGGACCCTGAGACACATT | | 60 | 129 |
| Ppia(F) | ATGGTCAACCCCACCGTGT | 25518 | 60 | 101 |
| Ppia(R) | TCTGCTGTCTTTGGAACTTTGTCT | | 60 | 101 |
